# Efficacy of Potassium-Competitive Acid Blockers Versus Proton Pump Inhibitors in Eradication Regimens for Clarithromycin-Resistant *Helicobacter pylori*: A Systematic Review and Meta-Analysis

**DOI:** 10.3390/pathogens15090986

**Published:** 2026-09-17

**Authors:** Dmitrii N. Andreev, Alsu R. Khurmatullina, Dmitry S. Bordin, Andrey V. Zaborovsky, Philipp S. Sokolov, Yury A. Kucheryavyy, Petr A. Beliy, Igor V. Maev

**Affiliations:** 1 Department of Internal Disease Propaedeutics and Gastroenterology, Russian University of Medicine, 127473 Moscow, Russia; 2 I.M. Sechenov First Moscow State Medical University, 119991 Moscow, Russia; 3 Department of Pancreatic, Biliary and Upper Digestive Tract Disorders, A. S. Loginov Moscow Clinical Scientific Center, 111123 Moscow, Russia; 4 Department of General Medical Practice and Family Medicine, Tver State Medical University, 170100 Tver, Russia; 5 Department of Pharmacology, Russian University of Medicine, 127473 Moscow, Russia; 6 Ilyinskaya Hospital, 143421 Krasnogorsk, Russia

**Keywords:** *Helicobacter pylori*, clarithromycin resistance, potassium-competitive acid blockers, P-CAB, vonoprazan, tegoprazan, eradication therapy, systematic review, meta-analysis

## Abstract

Background: Clarithromycin resistance represents the primary obstacle to the success of conventional proton pump inhibitor (PPI)-based triple therapy for Helicobacter pylori (*H. pylori*) eradication. Potassium-competitive acid blockers (P-CABs) offer faster and more potent acid suppression, which may enhance the efficacy of eradication regimens in this resistant population. The present study aimed to systematically evaluate the comparative efficacy of P-CAB-based versus PPI-based eradication regimens specifically in adult patients with documented clarithromycin-resistant *H. pylori* infection. Methods: This systematic review and meta-analysis was conducted in accordance with the PRISMA 2020 guidelines. The protocol was prospectively registered in PROSPERO (CRD420261407870). A systematic literature search was performed in Embase; MEDLINE/PubMed; CENTRAL; and Web of Science for articles published from 1 February 2015 to 31 March 2026. The included evidence comprised randomized and non-randomized comparative studies, as well as non-comparative studies. The primary outcome was the *H. pylori* eradication rate. Pooled risk ratios (RRs) with 95% confidence intervals (CIs) were calculated using a random-effects model (DerSimonian–Laird method). Risk of bias was assessed using RoB 2 for randomized comparative studies, ROBINS-I for non-randomized comparative studies, and the JBI Critical Appraisal Checklist for Case Series for non-comparative studies. Results: A total of 20 unique publications were included; 12 contributed data to the comparative analysis and 9 contributed data to the non-comparative single-arm analysis, with one publication contributing to both analyses. Across the 12 comparative studies, successful eradication was achieved in 464 of 621 patients (74.7%) receiving P-CAB-based regimens and in 301 of 571 patients (52.7%) receiving PPI-based comparators. P-CAB-based regimens were associated with a significantly higher probability of successful eradication compared with PPIs (RR, 1.42; 95% CI, 1.14–1.76; *p* < 0.00001). Subgroup analysis according to the P-CAB agent demonstrated that vonoprazan-based regimens exhibited the strongest comparative effect (RR, 1.78; 95% CI, 1.55–2.04), while tegoprazan-based regimens also remained superior (RR, 1.26; 95% CI, 1.04–1.54). The benefit of P-CABs was most pronounced in the clarithromycin-containing triple therapy subgroup (RR, 1.79; 95% CI, 1.55–2.07). Conclusions: P-CAB-based eradication regimens are significantly more effective than PPI-based comparators in patients with clarithromycin-resistant *H. pylori* infection. Further well-designed studies are warranted to determine the optimal antibiotic combinations, treatment durations, and regional applicability of these regimens.

## 1. Introduction

*Helicobacter pylori* (*H. pylori*) infection remains one of the most common chronic bacterial infections worldwide and is a major cause of chronic gastritis, peptic ulcer disease, mucosa-associated lymphoid tissue lymphoma, and gastric cancer [1,2]. Although its prevalence has declined over recent decades, the global burden remains substantial: a recent meta-analysis estimated that 43.9% of adults were infected with *H. pylori* in 2015–2022 [3]. Eradication therapy is therefore a key component of *H. pylori*-associated diseases and an important strategy for preventing long-term complications, particularly gastric cancer [2,4].

The success of eradication therapy is increasingly limited by antimicrobial resistance. Among resistance patterns, clarithromycin resistance is especially clinically relevant because it markedly reduces the efficacy of conventional clarithromycin-containing proton pump inhibitor (PPI)-based triple therapy [5]. Updated global analyses confirm that resistance remains widespread and geographically heterogeneous [6,7]. Clinically important levels of clarithromycin resistance have also been reported in Europe, the United States, Russia, and mainland China, underscoring the need for regionally informed treatment strategies [8,9,10].

Potassium-competitive acid blockers (P-CABs) are a newer class of antisecretory agents that are increasingly incorporated into *H. pylori* management. The Maastricht VI/Florence consensus and the updated American College of Gastroenterology guideline recognize P-CAB-based regimens as effective treatment options, while the American Gastroenterological Association has advised using P-CABs in place of PPIs in eradication regimens for most patients with *H. pylori* infection [2,4,11]. Compared with PPIs, P-CABs provide more rapid, potent, and sustained acid suppression, are less dependent on meal timing, and are less affected by CYP2C19-related variability [12,13].

These pharmacological advantages may translate into improved eradication outcomes. Several systematic reviews and meta-analyses have reported higher eradication rates with P-CAB-based regimens than with PPI-based regimens, although the magnitude of benefit varies according to the specific P-CAB, antibiotic regimen, treatment duration, and clinical setting [14,15,16]. This finding is consistent with resistant-subgroup data from a randomized clinical trial conducted in the United States and Europe and with an earlier focused meta-analysis of vonoprazan-based therapy [17,18]. More profound and sustained acid suppression may improve eradication efficacy in clarithromycin-resistant infection by maintaining a higher intragastric pH, thereby enhancing the stability and activity of co-administered antibiotics and promoting the bactericidal activity of amoxicillin against replicating *H. pylori* [19,20].

The previous meta-analysis specifically focused on clarithromycin-resistant infection was published in 2018 and was limited to vonoprazan-based regimens [17]. Since then, the evidence base has expanded beyond vonoprazan, notably to tegoprazan, and now includes a broader range of treatment regimens and resistance-assessment methods [18,21,22]. Therefore, an updated systematic review and meta-analysis is needed to evaluate the comparative efficacy of P-CAB-based versus PPI-based eradication regimens specifically in adults with documented clarithromycin-resistant *H. pylori* infection.

## 2. Materials and Methods

### 2.1. Search Strategy

This systematic review and meta-analysis was conducted in accordance with the Preferred Reporting Items for Systematic Reviews and Meta-Analyses statement (PRISMA 2020) [23]. The review protocol was developed prospectively and submitted for registration in PROSPERO (CRD420261407870). For further details, the full PRISMA checklist is available in Supplementary File S1.

A systematic literature search was performed in Embase, MEDLINE/PubMed, CENTRAL, and Web of Science. Databases were searched for articles published from 1 February 2015 to 31 March 2026. No language restrictions were applied. Only published studies were sought.

The search strategy combined controlled vocabulary and free-text terms related to *H. pylori*, P-CABs, PPIs, eradication therapy, and clarithromycin resistance. The following key terms and their variants were used: “(“Helicobacter pylori” [MeSH] OR “Helicobacter pylori” OR “H. pylori”) AND (“potassium-competitive acid blocker” OR “potassium competitive acid blocker” OR “P-CAB” OR “PCAB” OR “vonoprazan” OR “tegoprazan” OR “keverprazan” OR “fexuprazan” OR “revaprazan”) AND (“clarithromycin” [MeSH] OR “clarithromycin” OR “clarithromycin resistance” OR “clarithromycin-resistant” OR “23S rRNA” OR “A2142G” OR “A2143G” OR “susceptibility”) AND (“eradication” OR “eradication therapy” OR “treatment success” OR “treatment failure”). The complete search strategy is presented in Supplementary Table S1.

### 2.2. Eligibility Criteria

Studies were considered eligible if they met the following criteria: included adult patients with confirmed *H. pylori* infection and documented clarithromycin-resistant strains; evaluated a P-CAB-based eradication regimen; reported eradication outcomes separately for patients with clarithromycin-resistant infection. Comparative studies with an eligible PPI comparator were included in the comparative meta-analysis, whereas non-comparative P-CAB cohorts were included only in prespecified single-arm analyses of eradication rates. The review question was structured according to the PICO framework: adult patients with clarithromycin-resistant *H. pylori* infection; P-CAB-based eradication regimens as the intervention; PPI-based eradication regimens as the comparator; and *H. pylori* eradication rate as the primary outcome.

Clarithromycin resistance was defined according to the criteria used in the original studies, including phenotypic antimicrobial susceptibility testing, such as minimum inhibitory concentration testing, or molecular detection of clarithromycin-resistance-associated mutations, mainly 23S rRNA gene mutations. Studies were excluded if they did not provide separate data for clarithromycin-resistant patients, included pediatric populations, or did not report sufficient data to calculate eradication rates. Studies or treatment arms without an eligible PPI-based comparator were excluded from the pooled comparative effect estimate, as comparative effect estimates require data from both intervention and comparator groups. Such data were summarized and used in single-arm analyses of eradication rates.

### 2.3. Study Selection

After removal of duplicates, titles and abstracts were screened independently by two reviewers. Full texts of potentially eligible articles were then assessed against the predefined eligibility criteria. Disagreements at any stage were resolved by discussion or, when necessary, by consultation with a third reviewer. The study selection process was documented using a PRISMA flow diagram.

### 2.4. Data Extraction

Data were extracted independently by two reviewers (D.N.A. and A.R.K.) using a standardized extraction form. Conflicts were resolved by a third independent researcher (D.S.B.). The following information was collected from each eligible study: first author, year of publication, country, study design, sample size, patient characteristics, line of eradication therapy, method used to determine clarithromycin resistance, P-CAB and PPI regimens, antibiotic choice, treatment duration, eradication assessment method, and eradication outcomes.

For quantitative synthesis, the number of patients with successful eradication and the total number of treated patients were extracted separately for P-CAB-based and PPI-based regimens among clarithromycin-resistant patients. If multiple relevant treatment arms were reported within one study, eligible arms were extracted separately, taking care to avoid double-counting of comparator groups.

### 2.5. Statistical Analysis

For dichotomous outcomes, treatment effects were summarized as risk ratios (RRs) with 95% confidence intervals (CIs). The primary comparison was P-CAB-based versus PPI-based eradication therapy in patients with clarithromycin-resistant H. pylori infection. A random-effects model using the DerSimonian–Laird method was applied because clinical and methodological heterogeneity was expected across studies.

For single-arm meta-analyses of eradication proportions, pooled estimates were calculated using a binomial-normal random-effects generalized linear mixed model (GLMM) with a logit link. Summary estimates and corresponding 95% CIs were back-transformed to the proportion scale. Study-specific 95% CIs were calculated using exact binomial methods. This approach allowed studies with very high or complete eradication rates to be included without relying on the normal approximation for raw proportions.

Statistical heterogeneity was assessed using the Cochran Q test and the I^2^ statistic. I^2^ values of approximately 25%, 50%, and 75% were interpreted as low, moderate, and substantial heterogeneity, respectively. When sufficient data were available, subgroup analyses were planned according to P-CAB type, eradication regimen, treatment duration, line of therapy, method of clarithromycin resistance assessment, and study design.

Potential publication bias was planned to be assessed by visual inspection of funnel plots. *p* values < 0.05 were considered statistically significant. Statistical analyses were conducted using RevMan software (version 5.4.1; London, UK). Single-arm binomial-normal random-effects analyses were performed separately using a generalized linear mixed-effects modelling approach in Python 3.14 using SciPy software.

### 2.6. Risk of Bias Assessment

Risk of bias was assessed independently by two reviewers using design-specific tools. Randomized comparative studies were evaluated using the revised Cochrane risk-of-bias tool for randomized trials (RoB 2) [24]. Non-randomized comparative studies were assessed using the Risk Of Bias In Non-randomized Studies of Interventions (ROBINS-I) tool [25]. Non-comparative P-CAB cohorts contributing to the single-arm analyses were assessed using the Joanna Briggs Institute (JBI) Critical Appraisal Checklist for Case Series. Disagreements were resolved by consensus.

## 3. Results

**Figure 1:**
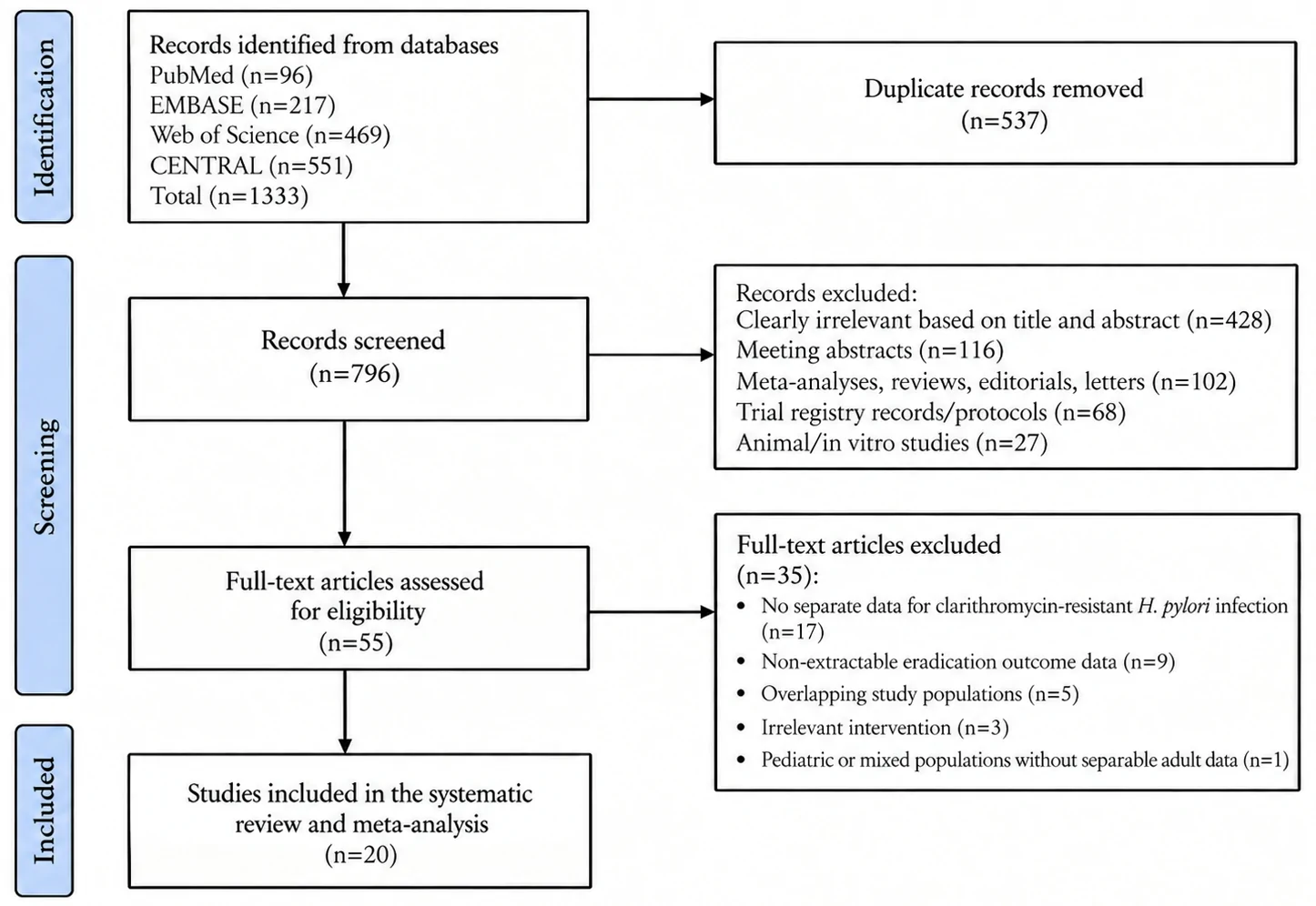
PRISMA flow-chart.

A total of 1333 records were identified through database searching, including 96 records from PubMed, 217 from EMBASE, 551 from CENTRAL, and 469 from Web of Science. After the removal of 537 duplicate records, 796 records remained for title and abstract screening. Of these, 741 records were excluded, including animal or in vitro studies, meeting abstracts, trial registry records or protocols, meta-analyses, reviews, editorials, letters, and studies that were clearly irrelevant based on title and abstract screening. The full texts of 55 articles were assessed for eligibility, and 35 articles were excluded after full-text review (Figure 1). The main reasons for exclusion were the absence of separate data for clarithromycin-resistant *Helicobacter pylori* infection, non-extractable eradication outcome data, overlapping study populations, irrelevant intervention, and pediatric or mixed populations without separable adult data. Finally, 20 unique publications contributing 21 eligible study datasets (Table 1 and Table 2) were included in the systematic review and meta-analysis [18,21,22,26,27,28,29,30,31,32,33,34,35,36,37,38,39,40,41,42].

### 3.1. Risk of Bias Evaluation

**Figure 2:**
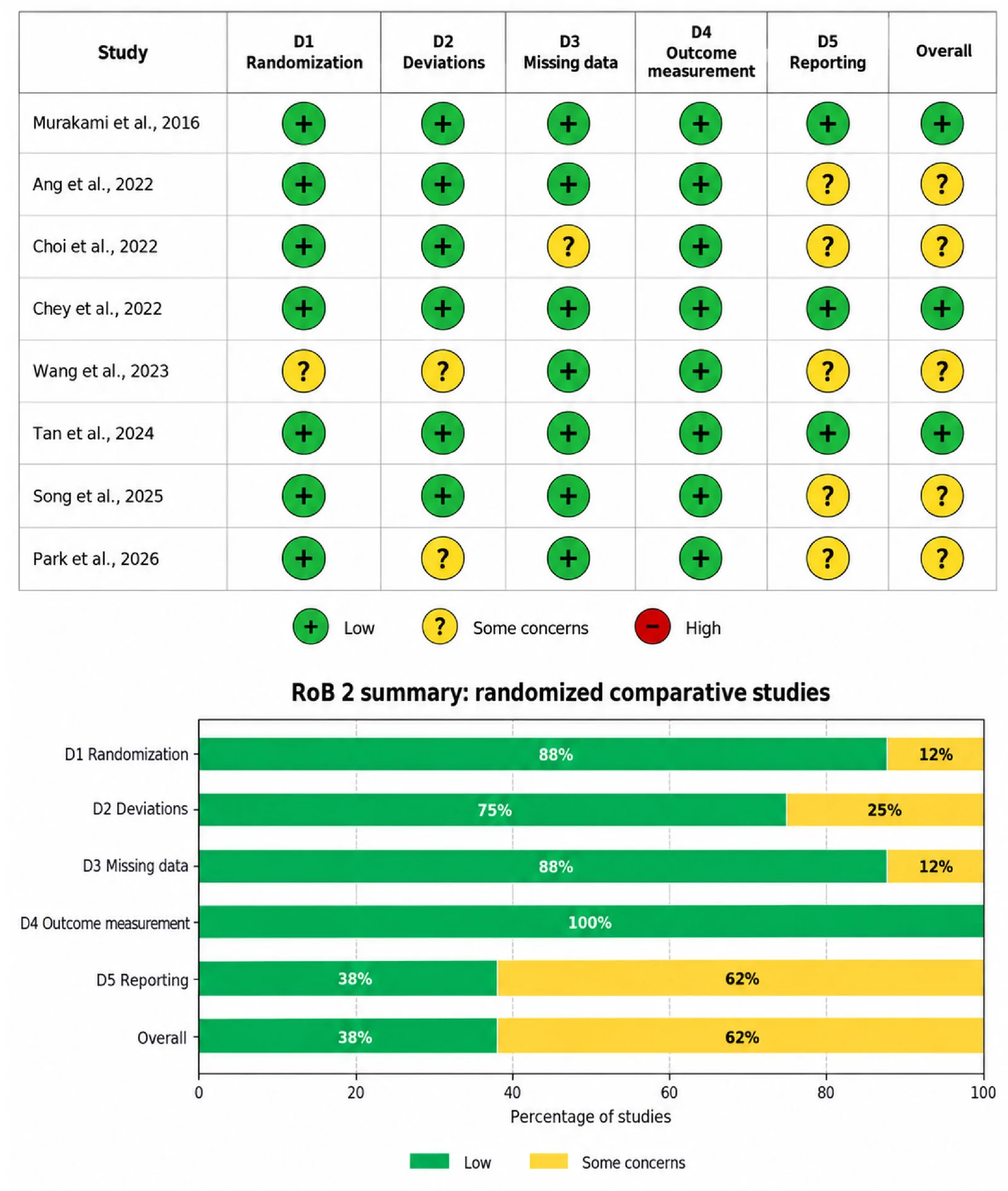
Risk-of-bias assessment of randomized comparative studies using RoB 2 [18,21,26,30,31,32,34,35].

Study quality was assessed separately according to study design. Among the eight randomized comparative trials, risk of bias was assessed using RoB 2. Overall risk of bias was generally low or raised some concerns, mainly because of incomplete reporting of allocation procedures, missing outcome data, or selective reporting in several studies (Figure 2).

**Figure 3:**
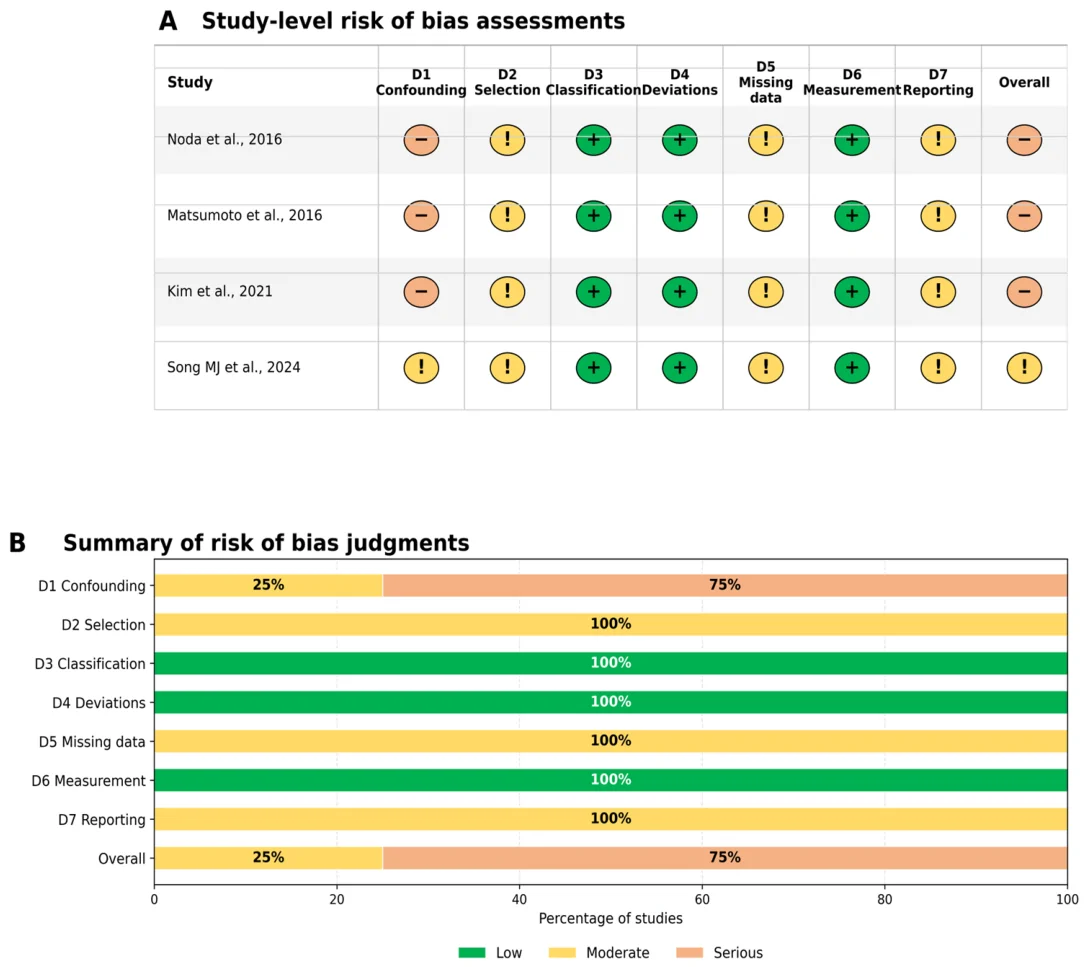
Risk-of-bias assessment of non-randomized comparative studies using ROBINS-I [27,28,29,33].

Four non-randomized comparative studies were assessed using ROBINS-I. The main concerns involved confounding, participant selection, missing data, and selective reporting, whereas classification of interventions, deviations from intended interventions, and outcome measurement were generally judged to be at low risk of bias. The detailed ROBINS-I assessment is presented in Figure 3.

The methodological quality of the nine publications contributing 12 non-comparative P-CAB cohorts to the single-arm analysis assessed using the JBI Checklist varied. Two studies were rated as high quality, four as moderate quality, and three as low quality. While outcome reporting was generally adequate, an important limitation was the unclear consecutive inclusion of participants in several studies. The detailed JBI Critical Appraisal Checklist for Case Series assessment is provided in Supplementary Table S2.

### 3.2. Meta-Analysis Results. Efficacy of P-CAB-Based Regimens in Clarithromycin-Resistant H. pylori Infection

#### 3.2.1. Comparative Efficacy of P-CAB-Based Versus PPI-Based Regimens

Twelve comparative studies were included. When all eligible comparative arms listed in Table 1 were considered, successful eradication was achieved in 464 of 621 patients receiving P-CAB-based regimens and in 301 of 571 patients receiving PPI-based comparators. For studies with more than one eligible P-CAB arm sharing the same PPI comparator, comparator group data were not double-counted in the pooled totals. The corresponding crude eradication rates were 74.7% and 52.7%, respectively.

**Figure 4:**
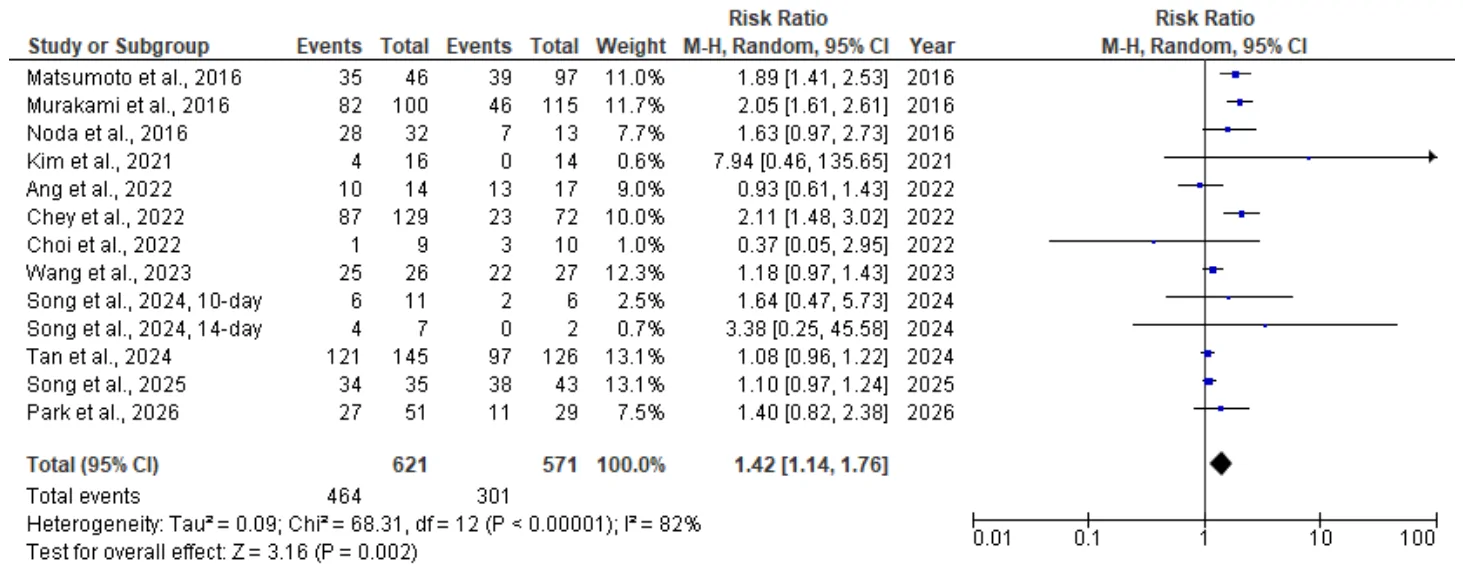
Eradication outcomes of comparative studies evaluating P-CAB-based versus PPI-based regimens in clarithromycin-resistant *H. pylori* infection [18,21,26,27,28,29,30,31,32,33,34,35].

**Figure 5:**
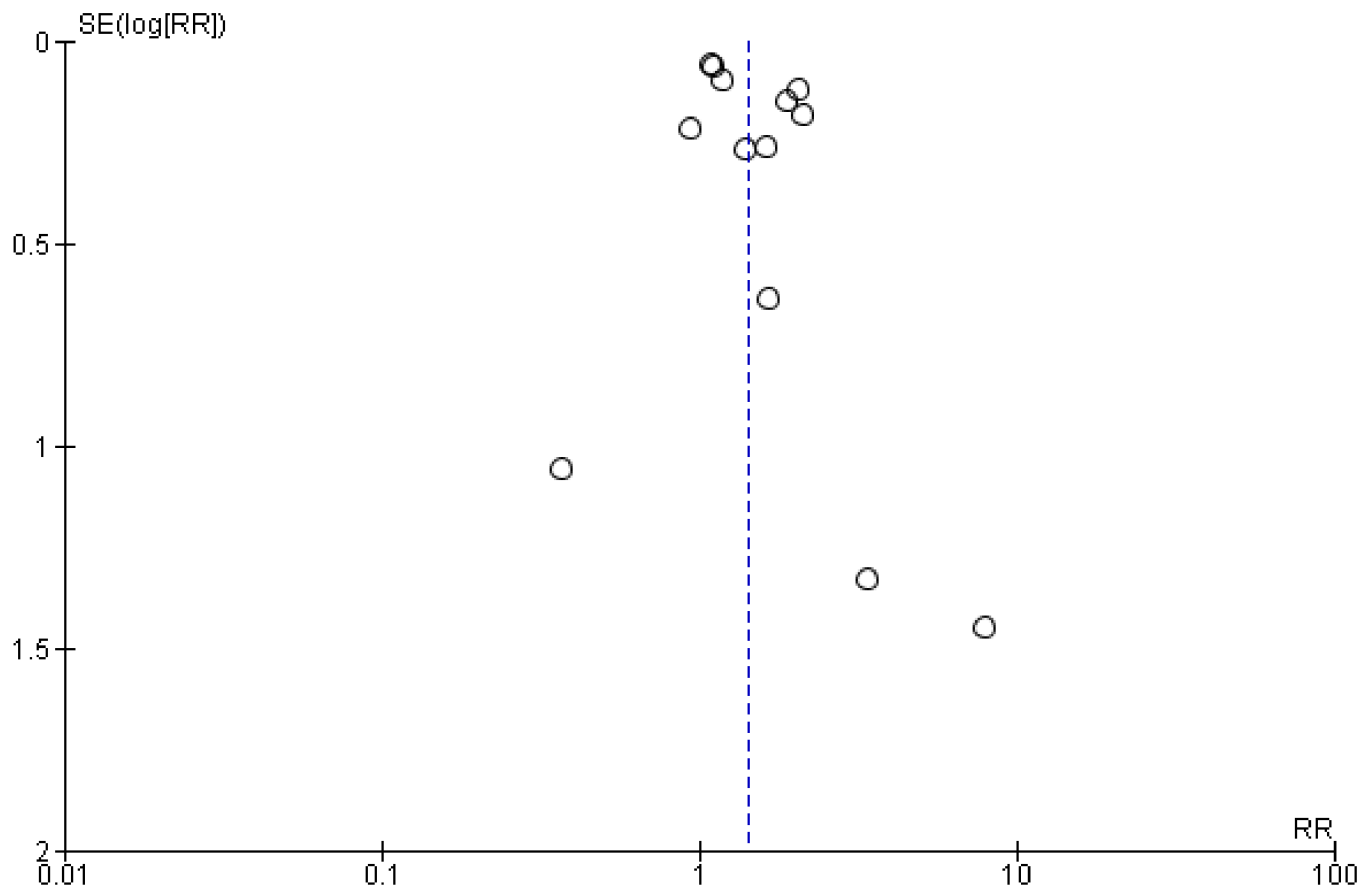
Funnel plot of comparative studies evaluating P-CAB-based versus PPI-based regimens.

In the random-effects meta-analysis, P-CAB-based regimens were associated with a significantly higher probability of successful eradication than PPI-based regimens (RR, 1.42; 95% CI, 1.14–1.76; *p* = 0.002). Substantial between-study heterogeneity was observed (I^2^ = 82%, *p* < 0.00001) (Figure 4). The funnel plot for the primary comparative analysis did not show substantial asymmetry, suggesting no clear evidence of small-study effects (Figure 5). However, given the limited number of comparative studies (n = 12), visual inspection of the funnel plot has low statistical power; therefore, the lack of observed asymmetry does not definitively exclude the presence of publication bias or small-study effects.

In an additional single-arm analysis restricted to P-CAB arms from comparative studies, the random-effects pooled eradication rate was 71.94% (95% CI, 56.92–83.26).

#### 3.2.2. Eradication Rates with Non-Comparative P-CAB-Based Regimens

**Figure 6:**
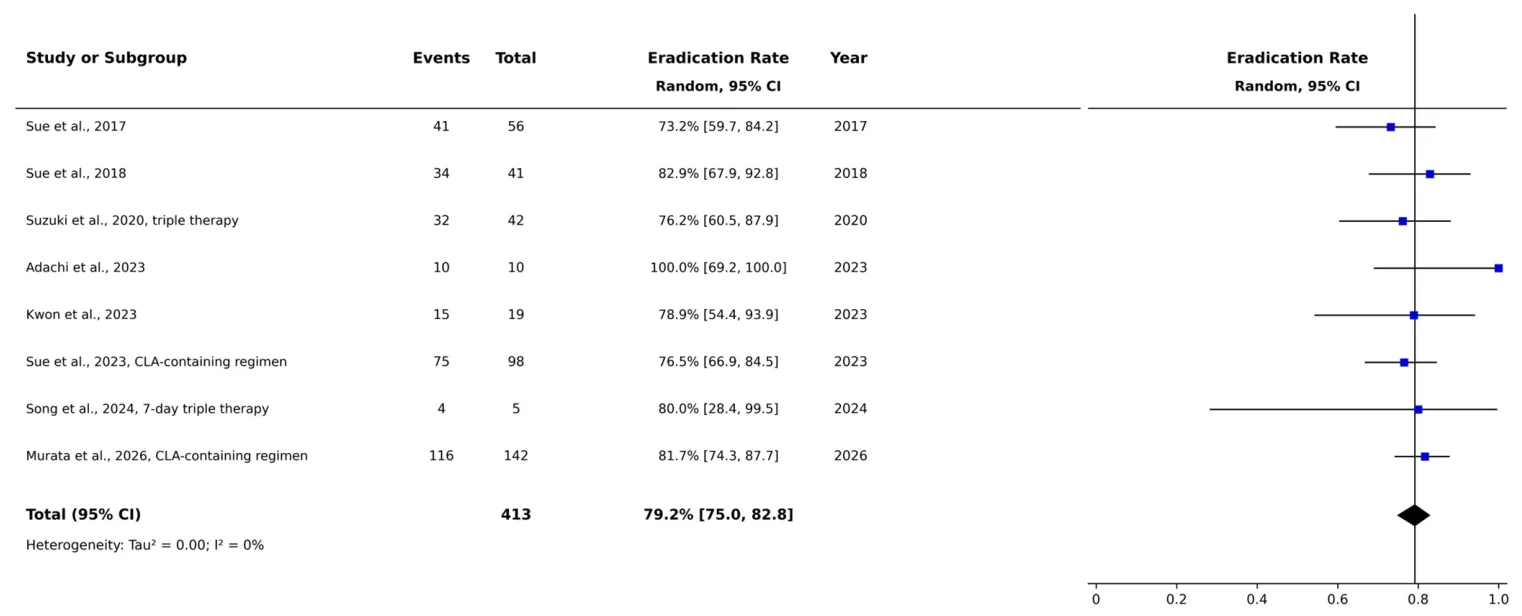
Restricted single-arm analysis of clarithromycin-containing P-CAB regimens [22,33,36,37,38,39,40,41].

Nine non-comparative studies comprising 12 eligible regimen cohorts were included. The random-effects pooled eradication rate was 88.86% (95% CI, 76.10–95.23%). High between-study heterogeneity was observed (I^2^ = 92%). Because several cohorts evaluated regimens that did not contain clarithromycin, an additional restricted analysis was performed including only clarithromycin-containing regimens. After exclusion of four non-clarithromycin-containing cohorts, eight eligible cohorts remained, comprising 413 patients (Figure 6). The pooled eradication rate was 79.18% (95% CI, 74.99–82.82).

### 3.3. Sensitivity Analysis

#### 3.3.1. Robustness Analysis According to Methodological Quality

Sensitivity analyses were performed to assess whether studies with lower methodological quality influenced the main findings. In the comparative analysis, after exclusion of studies judged to have high overall risk of bias or greater methodological concerns, 9 comparative studies were retained. The pooled effect remained in favor of P-CAB-based regimens, with an RR of 1.33 (95% CI, 1.06–1.67), which was comparable to the primary overall estimate (RR, 1.42; 95% CI, 1.14–1.76). The pooled eradication rate among the retained P-CAB arms was 73.18% (95% CI, 56.67–85.05), remaining within the same clinically relevant range as the primary pooled estimate. For the non-comparative analysis, studies judged to be at serious overall risk of bias were also excluded; this sensitivity analysis retained 6 studies, corresponding to 9 eligible P-CAB regimen cohorts. The pooled eradication rate in this sensitivity analysis was 89.85% (95% CI, 76.25–96.06). The exclusion of these lower-quality cohorts did not change the interpretation of the findings. Overall, these sensitivity analyses indicate that the observed benefit of P-CAB-based therapy was not driven by studies with greater risk-of-bias concerns.

#### 3.3.2. Sensitivity Analysis Excluding Small Clarithromycin-Resistant Subgroups

To assess the robustness of the findings to sparse data, we performed a post hoc sensitivity analysis excluding comparisons with a total clarithromycin-resistant subgroup size of 30 patients or fewer. The threshold of 30 patients was selected as an arbitrary cutoff to evaluate the impact of sparse data on the overall estimate. This analysis excluded the comparisons reported by Kim et al., Choi et al., and Song et al. The pooled effect estimate remained RR, 1.48; 95% CI, 1.38–1.61 I^2^ = 88%; *p* < 0.00001.

#### 3.3.3. Subgroup Analysis According to the P-CAB Agent

Among studies evaluating vonoprazan-based regimens, successful eradication was achieved in 267 of 347 patients receiving P-CAB-based therapy and in 150 of 341 patients receiving PPI-based comparators. Vonoprazan-based regimens were associated with a significantly higher probability of successful eradication than PPI-based regimens (RR, 1.78; 95% CI, 1.55–2.04; *p* < 0.00001). High between-study heterogeneity was observed (I^2^ = 82%; *p* < 0.0001).

When analyzing studies that evaluated tegoprazan-based regimens, successful eradication was achieved in 76 of 129 patients receiving P-CAB-based therapy and in 54 of 104 patients receiving PPI-based comparators. The pooled estimate favored tegoprazan-based regimens; the difference reached statistical significance (RR, 1.26; 95% CI, 1.04–1.54; *p* = 0.03). Between-study heterogeneity was moderate (I^2^ = 41%; *p* = 0.13).

#### 3.3.4. Subgroup Analysis According to the Antibiotic Regimen

Regimen-specific subgroup analyses were restricted to comparisons in which the accompanying antibiotics were matched between the P-CAB-based and PPI-based groups.

Among patients receiving clarithromycin-containing triple therapy, the pooled analysis showed a significantly greater likelihood of eradication with P-CAB-based therapy (RR, 1.79; 95% CI, 1.55–2.07; *p* < 0.00001). Heterogeneity across studies was moderate (I^2^ = 51%; *p* = 0.05).

In the subgroup of studies evaluating bismuth-containing quadruple therapy, the summary effect also indicated a statistically significant advantage of P-CAB-based regimens over PPI-based comparators (RR, 1.11; 95% CI, 1.01–1.23; *p* = 0.03), Table 3.

## 4. Discussion

Clarithromycin resistance remains one of the most clinically relevant causes of eradication failure, particularly when clarithromycin-containing PPI-based regimens are used empirically [2,4,5]. In this context, our meta-analysis specifically addressed whether replacing PPIs with P-CABs improves eradication outcomes in patients with documented clarithromycin-resistant *H. pylori* infection.

The main finding of this study is that P-CAB-based regimens were significantly more effective than PPI-based comparators in clarithromycin-resistant infection. In the primary comparative analysis, eradication was achieved in 464 of 621 patients receiving P-CAB-based therapy and in 301 of 571 patients receiving PPI-based therapy, corresponding to crude eradication rates of 74.7% and 52.7%, respectively. The pooled analysis confirmed a significant advantage of P-CAB-based regimens (RR, 1.42; 95% CI, 1.14–1.76; *p* < 0.00001). The sensitivity analysis excluding small clarithromycin-resistant subgroups yielded a consistent result, supporting the robustness of the overall finding.

The magnitude of benefit differed according to the individual P-CAB agent. Vonoprazan-based regimens showed the strongest comparative effect versus PPI-based therapy (RR, 1.78; 95% CI, 1.55–2.04); tegoprazan-based regimens also remained superior (RR, 1.26; 95% CI, 1.04–1.54). This suboptimal performance may not solely reflect pharmacokinetic differences, such as plasma half-life, but could also be heavily influenced by study-level factors. These may include shorter accompanying treatment durations (e.g., 7-day regimens compared to 14-day regimens), suboptimal dosing of the P-CAB or companion antibiotics, and potentially higher baseline patterns of secondary resistance (e.g., concurrent metronidazole resistance) within the specific cohorts evaluated for tegoprazan.

The regimen-specific analyses provide additional clinical context. The advantage of P-CABs was most evident in clarithromycin-containing triple therapy, where P-CAB-based regimens were associated with a markedly higher probability of eradication than matched PPI-based regimens (RR, 1.79; 95% CI, 1.55–2.07). A statistically significant but smaller benefit was observed in bismuth-containing quadruple therapy (RR, 1.11; 95% CI, 1.01–1.23). However, this finding should be interpreted with caution because the bismuth-containing subgroup was based on only a few heterogeneous studies. Thus, the smaller observed benefit may reflect limited evidence rather than a true difference in treatment effect. The additional restricted single-arm analysis further highlighted the importance of antibiotic composition. When the non-comparative analysis was limited to clarithromycin-containing P-CAB regimens, the pooled eradication rate decreased from 88.86% to 79.18% (95% CI, 74.99–82.82).

Our findings are consistent with previous evidence but extend it in several important ways. The earlier focused meta-analysis by Li et al. demonstrated the superiority of vonoprazan over PPIs for eradication of clarithromycin-resistant *H. pylori*, but it was limited to vonoprazan-based regimens and included a smaller evidence base [17]. More recently, Jin et al. reported eradication rates of 73.7% with P-CAB-based therapy versus 41.5% with PPI-based therapy in clarithromycin-resistant infection (RR, 1.53; 95% CI, 1.07–2.20), but this subgroup analysis was based on only three randomized studies and showed substantial heterogeneity [14]. The present analysis expands these observations by incorporating newer comparative and non-comparative evidence, including TPZ- and KPZ-based regimens, and by evaluating outcomes according to the individual P-CAB, antibiotic regimen, and susceptibility-testing method.

Broader meta-analyses of P-CAB-based therapy for *H. pylori* eradication, irrespective of clarithromycin susceptibility, have generally reported higher eradication rates with P-CAB-based regimens than with PPI-based regimens [15,16]. However, most of those analyses did not focus specifically on clarithromycin-resistant infection. Therefore, our results support the broader trend favoring P-CAB-based therapy while showing that the benefit is also present in the clinically challenging subgroup of patients with clarithromycin-resistant *H. pylori*.

Several mechanisms may explain the observed advantage of P-CAB-based therapy. Compared with conventional PPIs, P-CABs provide faster, more potent, and more sustained acid suppression, leading to higher and more stable intragastric pH during eradication therapy [43,44,45]. This pharmacodynamic advantage has been shown in randomized crossover studies of vonoprazan versus PPIs and is summarized in recent reviews of P-CABs in acid-related diseases and *H. pylori* infection [46]. A higher intragastric pH may improve the stability and activity of co-administered antibiotics and may be particularly relevant for amoxicillin-based regimens, since amoxicillin is most effective against actively replicating *H. pylori* [47]. Importantly, P-CABs do not reverse clarithromycin resistance itself; rather, they may reduce its clinical impact by optimizing the pharmacodynamic environment of eradication therapy and increasing the contribution of companion antibiotics, particularly amoxicillin [48].

An important nuance in resistance assessment is the distinction between microbiological resistance and clinical treatment failure [2]. The detection of clarithromycin-resistance-associated mutations (genotypic resistance, such as A2142G/A2143G point mutations in the 23S rRNA gene) indicates a biological mechanism of resistance, but it does not invariably lead to clinical treatment failure [49]. Phenotypic methods (e.g., MIC) account for the actual expression of resistance. In some cases, strains harboring specific point mutations may still exhibit MICs low enough to allow for eradication, particularly when the potent and sustained acid suppression provided by P-CABs maximizes the bactericidal activity of companion antibiotics like amoxicillin [45]. Thus, successful clinical eradication in the presence of a resistance mutation does not imply that the mutation failed to confer biological resistance; rather, it reflects that the optimized pharmacodynamic environment of the P-CAB-based regimen was sufficient to overcome the resistance mechanism [50].

P-CAB-based regimens should be considered within the broader therapeutic framework for clarithromycin-resistant *H. pylori* infection. Contemporary evidence supports the use of bismuth-containing quadruple therapy as an effective empirical option, although its performance may vary according to regional resistance patterns, antibiotic composition, and treatment duration [51]. This interpretation is consistent with our recent umbrella review of systematic reviews and meta-analyses, which showed that susceptibility-guided tailored therapy was superior to empirical therapy overall and particularly in first-line *H. pylori* eradication, although its advantage was less consistent in later treatment lines and depended on the empirical comparator used [8]. Susceptibility-guided therapy may improve eradication compared with older empirical strategies, but its incremental benefit appears less consistent when modern quadruple regimens are used [52]. Other strategies include optimized high-dose PPI–amoxicillin dual therapy, levofloxacin-based regimens when fluoroquinolone resistance is low, and rifabutin-containing therapy as a rescue option [53,54,55].

Umbrella-level evidence demonstrates that rebamipide improves *H. pylori* eradication efficacy, and real-world data from the European Registry on Helicobacter pylori Management, obtained in Russia where clarithromycin resistance exceeds 15%, show superior first-line empirical eradication rates with rebamipide-containing regimens [56,57,58]. In addition, an increasing body of evidence suggests that probiotics may also contribute to improved eradication outcomes in the setting of antibiotic resistance; however, their role requires further validation in well-designed studies before firm conclusions can be made [59].

Recent Asian guidelines further support the integration of P-CABs into contemporary *H. pylori* treatment algorithms. The 2024 revised Japanese guideline recommends antimicrobial susceptibility testing before eradication therapy and recognizes VPZ-based regimens as central options in first-line and tailored treatment strategies; in clarithromycin-resistant infection, VPZ–amoxicillin–metronidazole, VPZ–amoxicillin–sitafloxacin, or VPZ–amoxicillin dual therapy may be selected depending on metronidazole and quinolone susceptibility [60]. Similarly, the 2025 Korean guideline framework reflects a shift away from empirical clarithromycin-containing triple therapy in a setting of high clarithromycin resistance, emphasizing empirical quadruple regimens and PCR-guided tailored therapy, with P-CABs and bismuth-based regimens as key components [61].

Finally, treatment adherence remains a key determinant of eradication success. Even the most rationally selected regimen may fail if the patient does not complete therapy, whereas poor adherence may compromise eradication despite antimicrobial susceptibility [62]. This is particularly important in populations with substantial antibiotic resistance, where suboptimal drug exposure may further contribute to treatment failure and selection of secondary resistance [63]. Therefore, patient education, simplified dosing when possible, proactive management of adverse events, and adherence-support interventions should be regarded as essential components of eradication strategy rather than optional supportive measures [64].

Thus, P-CABs may improve eradication outcomes in clarithromycin-resistant infection, but they should complement, rather than replace, rational antibiotic selection when resistance data are available.

This study has several limitations. First, despite the inclusion of recent evidence, the number of studies remained limited in several subgroups, particularly for TPZ and KPZ. This smaller evidence base compared to vonoprazan results in wider confidence intervals and lower statistical power for these newer agents. Second, most data came from regions where P-CABs are already available, which may limit generalizability to other geographic settings. Third, substantial heterogeneity observed in the primary analysis indicates that the magnitude of the relative benefit of P-CAB-based therapy may vary across clinical settings. Potential contributors include differences in antibiotic combinations, treatment duration, treatment line, study design, geographic setting, and methods used to identify clarithromycin resistance. Therefore, the pooled RR should be interpreted as an average treatment effect across heterogeneous clinical contexts rather than as a uniform effect applicable to all P-CAB regimens and patient populations. Fourth, several clinically important regimens, including optimized bismuth quadruple therapy and high-dose dual therapy, were not adequately represented. Finally, some analyses were based on relatively small clarithromycin-resistant subgroups.

The strengths of this review include its specific focus on clarithromycin-resistant *H. pylori* infection, inclusion of updated evidence beyond vonoprazan, and differentiated analyses according to the individual P-CAB agent, antibiotic regimen, and susceptibility-testing method. These features provide a more clinically targeted assessment of the role of P-CAB-based therapy in a population where conventional PPI-based clarithromycin-containing regimens are particularly vulnerable to failure.

## 5. Conclusions

In patients with clarithromycin-resistant *H. pylori* infection, P-CAB-based eradication regimens were associated with higher eradication rates than PPI-based comparators. Further well-designed studies are needed to define the optimal P-CAB agent, antibiotic combination, treatment duration, and regional applicability of these regimens.

## Figures and Tables

**Table 1:** Comparative studies of P-CAB-based versus PPI-based regimens.

Study	P-CAB-Based Regimen	PPI-Based Comparator	CLA-R Detection Method	P-CAB Eradication, n/N	PPI Eradication, n/N
Murakami et al., 2016 [26]	VPZ + AMX + CLA	LAN + AMX + CLA	MIC	82/100	46/115
Noda et al., 2016 [27]	VPZ + AMX + CLA	PPI (OME/LAN/ESO/RAB) + AMX + CLA	MIC	28/32	7/13
Matsumoto et al., 2016 [28]	VPZ + AMX + CLA	PPI (LAN/ESO/RAB) + AMX + CLA	23S rRNA mutation analysis	35/46	39/97
Kim et al., 2021 [29]	TPZ + AMX + CLA + Bi	LAN + AMX + CLA + Bi	23S rRNA mutation analysis	4/16	0/14
Ang et al., 2022 [30]	VPZ + AMX + CLA	PPI (OME/ESO/RAB) + AMX + CLA	MIC	10/14	13/17
Choi et al., 2022 [18]	TPZ + AMX + CLA	LAN + AMX + CLA	MIC	1/9	3/10
Chey et al., 2022 [21]	VPZ + AMX	LAN + AMX + CLA	23S rRNA mutation analysis	39/56	23/72
	VPZ + AMX + CLA			48/73	
Wang et al., 2023 [31]	VPZ + AMX	RAB + AMX + CLA + Bi	23S rRNA mutation analysis	25/26	22/27
Tan et al., 2024 [32]	KPZ + AMX + CLA + Bi	ESO + AMX + CLA + Bi	NR	121/145	97/126
Song et al., 2024, 14-day triple therapy [33]	TPZ + AMX + CLA	RAB + AMX + CLA	23S rRNA A2142G/A2143G mutation analysis	4/7	0/2
Song et al., 2024, 10-day concomitant therapy [33]	TPZ + AMX + CLA + MTZ	RAB + AMX + CLA + MTZ		6/11	2/6
Song et al., 2025 [34]	TPZ + AMX + CLA + Bi	ESO + AMX + CLA + Bi	MIC	34/35	38/43
Park et al., 2026 [35]	TPZ + AMX + CLA	LAN + AMX + CLA	23S rRNA mutation analysis	27/51	11/29

Abbreviations: AMX, amoxicillin; Bi, bismuth; CLA, clarithromycin; CLA-R, clarithromycin-resistant; ESO, esomeprazole; KPZ, keverprazan; LAN, lansoprazole; MIC, minimum inhibitory concentration; OME, omeprazole; P-CAB, potassium-competitive acid blocker; PPI, proton pump inhibitor; RAB, rabeprazole; TPZ, tegoprazan; VPZ, vonoprazan; 23S rRNA, 23S ribosomal RNA.

**Table 2:** Non-comparative P-CAB cohorts.

Study	P-CAB-Based Regimen	CLA-R Detection Method	Eradication, n/N
Sue et al., 2017 [36]	VPZ + AMX + CLA	MIC	41/56
Sue et al., 2018 [37]	VPZ + AMX + CLA	MIC	34/41
Suzuki et al., 2020, dual therapy [38]	VPZ + AMX	MIC	24/26
Suzuki et al., 2020, triple therapy [38]	VPZ + AMX + CLA	MIC	32/42
Adachi et al., 2023 [39]	VPZ + CLA + MTZ	MIC	10/10
Kwon et al., 2023 [40]	TPZ + AMX + CLA + MTZ	MIC	15/19
Sue et al., 2023, CLA-containing regimen [41]	VPZ + AMX + CLA	MIC	75/98
Sue et al., 2023, MTZ-containing regimen [41]	VPZ + AMX + MTZ	MIC	35/35
Song et al., 2024, 7-day triple therapy [33]	TPZ + AMX + CLA	23S rRNA A2142G/A2143G mutation analysis	4/5
Cho et al., 2025 [42]	TPZ + AMX	RT-PCR (A2142G/A2143G point mutations)	29/50
Murata et al., 2026, CLA-containing regimen [22]	VPZ + AMX + CLA	MIC	116/142
Murata et al., 2026, MTZ-containing regimen [22]	VPZ + AMX + MTZ	MIC	140/140

**Table 3:** Summary of primary, subgroup, and sensitivity analyses.

Analysis	Subgroup	Number of Studies	RR (95% CI)	I^2^	*p*-Value
Primary analysis	All comparative studies	12	1.42 (1.14–1.76)	82%	0.002
P-CAB agent	VPZ-based regimens	6	1.78 (1.55–2.04)	82%	<0.00001
	TPZ-based regimens	5	1.26 (1.04–1.54)	41%	0.03
	KPZ-based regimens	1	NR	NR	NR
Antibiotic regimen	CLA-containing triple therapy	8	1.79 (1.55–2.07)	51%	<0.00001
	Bismuth-containing quadruple therapy	3	1.11 (1.01–1.23)	4%	0.03
	P-CAB dual-therapy comparisons	2	1.72 (1.35–2.18)	94%	<0.00001
Susceptibility testing method	Phenotypic testing: MIC or culture-based methods	5	1.30 (1.18–1.42)	85%	<0.00001
	Genotypic testing: 23S rRNA mutation analysis	6	1.73 (1.45–2.06)	72%	<0.00001
Sensitivity analysis	Excluding CLA-R subgroups with ≤30 patients	9	1.48 (1.38–1.61)	88%	<0.00001

## Data Availability

Data is provided within the manuscript or Supplementary Information File.

## Supplementary Materials

The following supporting information can be downloaded at https://www.mdpi.com/article/10.3390/pathogens15090986/s1. Supplementary Table S1: Search strategy for each database; Supplementary Table S2: JBI checklist; Supplementary File S1: PRISMA 2020 Checklist.

## Abbreviations

The following abbreviations are used in this manuscript:

23S rRNA	23S ribosomal RNA
ACG	American College of Gastroenterology
AMX	amoxicillin
Bi	bismuth
CENTRAL	Cochrane Central Register of Controlled Trials
CI	confidence interval
CLA	clarithromycin
CLA-R	clarithromycin-resistant
CYP2C19	cytochrome P450 2C19
ESO	esomeprazole
H. pylori	Helicobacter pylori
Hp-EuReg	European Registry on Helicobacter pylori Management
KPZ	keverprazan
LAN	lansoprazole
MeSH	Medical Subject Headings
MIC	minimum inhibitory concentration
MTZ	metronidazole
NR	not reported
OME	omeprazole
P-CAB	potassium-competitive acid blocker
PICO	population, intervention, comparator, and outcome
PPI	proton pump inhibitor
PRISMA	Preferred Reporting Items for Systematic Reviews and Meta-Analyses
PROSPERO	International Prospective Register of Systematic Reviews
RAB	rabeprazole
RCT	randomized controlled trial
RoB 2	revised Cochrane risk-of-bias tool for randomized trials
RR	risk ratio
RT-PCR	real-time polymerase chain reaction
TPZ	tegoprazan
VPZ	vonoprazan
