# Supplementary material for: Efficacy of Potassium-Competitive Acid Blockers Versus Proton Pump Inhibitors in Eradication Regimens for Clarithromycin-Resistant Helicobacter pylori: A Systematic Review and Meta-Analysis
Source: Pathogens. 2026 Sep 17;15(9):986. doi: 10.3390/pathogens15090986 (PMC13610895; doi:10.3390/pathogens15090986)
Supplement: Supplementary file 1 [file pathogens-15-00986-s001.zip › JBI checklist.pdf]

Study	Q1: Inclusion Criteria	Q2: Standard Measurement	Q3: Valid Condition ID	Q4: Consecutive Inclusion	Q5: Complete Inclusion	Q6: Demographics Reported	Q7: Clinical Info Reported	Q8: Outcomes / Follow-up	Q9: Site / Clinic Info	Q10: Appropriate Statistics	Overall JBI Quality
**Sue et al., 2017**	Unclear	Yes	Yes	Unclear	Unclear	Yes	Yes	Yes	Unclear	Unclear	**Moderate**
**Sue et al., 2018**	Yes	Unclear	Yes	Unclear	Unclear	Yes	Yes	Yes	Unclear	Unclear	**Moderate**
**Suzuki et al., 2020** *(dual/triple)*	Unclear	Yes	Yes	Unclear	Unclear	Yes	Yes	Yes	Unclear	Unclear	**Moderate**
**Adachi et al., 2023**	No	Yes	Yes	No	No	Unclear	Unclear	Unclear	No	No	**Low**
**Kwon et al., 2023**	Unclear	Yes	Yes	Unclear	Unclear	Yes	Yes	Yes	Unclear	Unclear	**Moderate**
**Sue et al., 2023** *(CLA/MTZ)*	Yes	Yes	Yes	Yes	Yes	Yes	Yes	Yes	Yes	Yes	**High**
**Song et al., 2024** *(7-day triple)*	No	Yes	Yes	No	No	Unclear	Unclear	Unclear	No	No	**Low**
**Cho et al., 2025**	Unclear	Yes	Yes	No	Unclear	Unclear	Unclear	Unclear	Unclear	Unclear	**Low**
**Murata et al., 2026** *(CLA/MTZ)*	Yes	Unclear	Yes	Yes	Yes	Yes	Yes	Yes	Yes	Yes	**High**
