# Supplementary material for: Efficacy of Potassium-Competitive Acid Blockers Versus Proton Pump Inhibitors in Eradication Regimens for Clarithromycin-Resistant Helicobacter pylori: A Systematic Review and Meta-Analysis
Source: Pathogens. 2026 Sep 17;15(9):986. doi: 10.3390/pathogens15090986 (PMC13610895; doi:10.3390/pathogens15090986)
Supplement: Supplementary file 1 [file pathogens-15-00986-s001.zip › Table S1 Search strategy for each datab.pdf]

Supplementary Table S1. Detailed search strategy

Database	Search strategy
**MEDLINE (via PubMed)**	("Helicobacter pylori"[Mesh] OR "Helicobacter pylori" OR "H. pylori") AND ("Potassium-Competitive Acid Blockers"[Supplementary Concept] OR "potassium-competitive acid blocker*" OR "potassium competitive acid blocker*" OR P-CAB* OR PCAB* OR vonoprazan OR tegoprazan OR keverprazan OR fexuprazan OR revaprazan) AND ("Clarithromycin"[Mesh] OR clarithromycin OR "clarithromycin resistance" OR "clarithromycin-resistant" OR susceptibility OR "23S rRNA" OR A2142G OR A2143G) AND (eradication OR "eradication therapy" OR "treatment success" OR "treatment failure")
**Embase (Elsevier)**	('helicobacter pylori'/exp OR 'helicobacter pylori' OR 'h. pylori') AND ('potassium competitive acid blocker'/exp OR 'potassium competitive acid blocker*' OR 'potassium-competitive acid blocker*' OR p-cab* OR pcab* OR vonoprazan OR tegoprazan OR keverprazan OR fexuprazan OR revaprazan) AND ('clarithromycin'/exp OR clarithromycin OR 'clarithromycin resistance' OR 'clarithromycin resistant' OR susceptibility OR '23S rRNA' OR A2142G OR A2143G) AND (eradication OR 'eradication therapy' OR 'treatment success' OR 'treatment failure')
**Cochrane CENTRAL**	("Helicobacter pylori" OR "H. pylori") AND ("potassium-competitive acid blocker*" OR "potassium competitive acid blocker*" OR P-CAB* OR PCAB* OR vonoprazan OR tegoprazan OR keverprazan OR fexuprazan OR revaprazan) AND (clarithromycin OR "clarithromycin resistance" OR "clarithromycin-resistant" OR susceptibility OR "23S rRNA" OR A2142G OR A2143G) AND (eradication OR "eradication therapy" OR "treatment success" OR "treatment failure")
**Web of Science**	TS=("Helicobacter pylori" OR "H. pylori") AND TS=("potassium-competitive acid blocker*" OR "potassium competitive acid blocker*" OR P-CAB* OR PCAB* OR vonoprazan OR tegoprazan OR keverprazan OR fexuprazan OR revaprazan) AND TS=(clarithromycin OR "clarithromycin resistance" OR "clarithromycin-resistant" OR susceptibility OR "23S rRNA" OR A2142G OR A2143G) AND TS=(eradication OR "eradication therapy" OR "treatment success" OR "treatment failure")
